# Autoregressive count data modeling on mobility patterns to predict cases of COVID-19 infection

**DOI:** 10.1007/s00477-022-02255-6

**Published:** 2022-06-23

**Authors:** Jing Zhao, Mengjie Han, Zhenwu Wang, Benting Wan

**Affiliations:** 1 School of Business Administration, Xi’an Eurasia University, Yanta District, Xi’an, China; 2 School of Information and Engineering, Dalarna University, 79188 Falun, Sweden; 3 Department of Computer Science and Technology, China University of Mining and Technology, Beijing, 100083 China; 4 School of Software and IoT Engineering, Jiangxi University of Finance and Economics, Nanchang, 330013 China

**Keywords:** COVID-19, Mobility, Generalized linear model, Autoregressive model, Quasi-likelihood

## Abstract

At the beginning of 2022 the global daily count of new cases of COVID-19 exceeded 3.2 million, a tripling of the historical peak value reported between the initial outbreak of the pandemic and the end of 2021. Aerosol transmission through interpersonal contact is the main cause of the disease’s spread, although control measures have been put in place to reduce contact opportunities. Mobility pattern is a basic mechanism for understanding how people gather at a location and how long they stay there. Due to the inherent dependencies in disease transmission, models for associating mobility data with confirmed cases need to be individually designed for different regions and time periods. In this paper, we propose an autoregressive count data model under the framework of a generalized linear model to illustrate a process of model specification and selection. By evaluating a 14-day-ahead prediction from Sweden, the results showed that for a dense population region, using mobility data with a lag of 8 days is the most reliable way of predicting the number of confirmed cases in relative numbers at a high coverage rate. It is sufficient for both of the autoregressive terms, studied variable and conditional expectation, to take one day back. For sparsely populated regions, a lag of 10 days produced the lowest error in absolute value for the predictions, where weekly periodicity on the studied variable is recommended for use. Interventions were further included to identify the most relevant mobility categories. Statistical features were also presented to verify the model assumptions.

## Introduction

It has been two years since a group of patients with pneumonia were found in Wuhan, China (Zhu et al. 2020). While displaying symptoms similar to the severe acute respiratory syndrome (SARS) (Zhou et al. 2020a), upon investigation it was discovered that they had contracted a novel form of a coronavirus, named SARS-CoV-2. The disease was later named COVID-19 (WHO 2020). The basic reproduction number ${R}_{0}$ is a measurement of the average number of secondary cases that one primary case will generate. For COVID-19 this number was estimated to be 2.2 in western Europe and 3.38 in China (Alimohamadi et al. 2020; Locatelli et al. 2021). The ${R}_{0}$ s of the Delta and Omicron variants have been higher compared to the ancestral SARS-CoV-2 virus (Liu and Rocklöv 2021; Nishiura et al. 2021). There is currently no medication approved by US Food and Drug Administration that offers a cure for this disease (Güner et al. 2020). The death rate for COVID-19 has been reported as 3.4%, but this figure can vary depending on factors such as age, sex, the overall health of a population and the extent or otherwise of a national health system. For example, for those with cardiovascular disease the death rate from COVID-19 is 10.5% while for diabetes it is 7.3% (Ahmad 2020). Without effective control measures, regions with relatively older populations could see disproportionally more cases (Davies et al. 2020). Most countries struggle to control the social and economic activities which lie behind many large-scale gatherings, thus making it difficult to isolate the sources of infection and cut off the channels of transmission. The primary control measures continue to be good hand hygiene, social distancing and quarantine. Any additional preventative measures should consider the local environment and be country dependent.

Large-scale governmental measures, such as national lockdowns and the widespread cancellation of mass gatherings, may be taken quickly and produce instant results. However, the implementation of these strategies is usually *ex post facto* leaving limited time for organizations and the general public to adjust. In contrast, model-based predictions could enable governments to announce soft control measures and take action well in advance. Based on accurate predictions, well-planned control measures could lessen the number of infected individuals as well as minimizing the costs of preventative measures (Mandal et al. 2020). In addition, control measures could provide the medical system with sufficient time to allocate resources. Models that can handle government interventions are preferred in actual decision-making. A dynamic loop between modeling and measures deployment could be created to establish an equilibrium point, where additional efforts made on improving a model’s accuracy will not radically affect a measure’s enaction. Thus, predictive models that help to understand the future trends of confirmed COVID-19 cases should serve as the basis for decision support at a national or regional level. The SARS-CoV-2 spreads primarily through respiratory droplets and close contact. Given that the most probable transmission pathway is between humans, even asymptomatic ones (Vella et al. 2020), of the best ways to chart its spread is by examining the variable of human mobility and the different patterns that can take. Mobility data has two key features: how people move and how long people stay at a place. Due to the characteristics of the data and the reduced opportunities for public transport (see Sect. 4.1), we only considered the second feature, mobility change at particular locations, to build the models in this paper.

Adopting mobility could generate some uncertainties in the modeling, for example, the choice of lag effect, the impact of population density and the identification of autoregressive terms. In order to handle these uncertainties, the aim of this paper is to investigate how mobility change can be effectively used to predict the number of cases of COVID-19 infection. The empirical findings are illustrated by using a set of regions, taken from a Nordic country, in this case Sweden, with heterogeneous population densities. The modeling framework is thought to be replicable in a similar context. The specific contributions that this paper aims to make are to:

1. Propose an autoregressive modeling framework for count data in regions with different population densities;
2. Identify the categories of locations where mobility change is decisive for accurate predictions;
3. Provide a scheme to deal with the lag effects and autoregressive terms;
4. Analyze the effect on modeling by including an intervention;
5. Present statistical characteristics for two distributions of the count data: Poisson and negative binomial.

The rest of the paper is organized as follows. Section 2 briefly reviews the research that has studied mobility as a means of analyzing COVID-19. Section 3 presents the generalized linear model for the autoregressive count data and the quasi-likelihood for parameter estimation. Section 4 outlines the nature of both the mobility data and the daily confirmed cases of COVID-19 in Sweden. Modeling implications and model applicability based on optimization results and predictions are given in Sect. 5. The last two sections discuss and conclude the work as well as point out future work directions.

## Existing research on mobility patterns and COVID-19

The modeling methods and data resources used for understanding the spread of COVID-19 are important components for building a framework for prediction. The same method may produce different results on different datasets. To provide a basis for the modeling approach taken in this paper, this section will provide a brief overview of the research on these components, as well as a short discussion of the lag effect.

### Empirical models for forecasting

Certain control measures, such as the suspension of transport infrastructures and the placing of widespread bans on public gatherings, can ensure a significant degree of social distancing that can have a knock-on effect on disease transmission (Tian et al. 2020; Vannoni et al. 2020). However, the effect of these measures can vary between region and time period, making them stochastic factors in the accurate and long-term prediction of COVID-19 cases. Mobility pattern, on the other hand, is a more sensible proxy for the interpersonal contact rates that contribute to COVIDd-19 transmission because it not only reflects the consequences of measure execution, but also quantifies how people gather and move. Reduced mobility can simultaneously reduce the peak number of cases and delay the peak, which could help to alleviate the demand burden in the medical system. For example, a 20% reduction in mobility can cut down by 33% the peak number of cases and delay the arrival of that peak by almost two weeks. The reduction of the peak and the delay in its arrival could reach 91% and 14 weeks with a 60% reduction in mobility (Zhou et al. 2020b).

There are many data-driven models that associate mobility patterns to case prediction. Basic statistical models that are easy to implement, such as multiple regression, least absolute shrinkage and selection operator, as well as ridge regression (LASSO), have been tested for short-term prediction (Wang et al. 2021). As the number of predicted days-head increased, however, the number of errors grew. Another simple and transparent statistical model that has been tried was to estimate the effect of non-pharmaceutical interventions on mobility by using basic machine learning methods to generate a 10-day ahead forecast. The indications are that mobility data on its own is sufficient to forecast meaningfully at all geographic scales (Ilin et al. 2021). Similarly, basic machine learning methods, such as regression tree, random forest and artificial neural network, have been combined in a linear model to deal with data in a non-linear relationship and with a non-normal distribution (Kuo and Fu 2021). The authors recommended that more sophisticated models for better prediction accuracy need to be explored.

Some efforts have been made to capture inherent temporal dependencies and spatial correlations. Using a LSTM network, for example, the combination of mobility and meteorological data was found to be the primary factor in case prediction (Rashed and Hirata 2021). A susceptible to infectious transition rate, as a function of mobility and social behavior with time-dynamics parameters, was modeled by a deep LSTM, which was further integrated into a susceptible-exposed-infectious-recovered (SEIR) model for case forecast (Bhouri et al. 2021). A graph neural network is another attempt to capture the spatio-temporal dynamics, where spatial edges represent mobility-based inter-region connectivity and temporal edges represent node features through time (Kapoor et al. 2020).

### Lag effect

A crucial issue in modeling is to determine the lag in days between the mobility data and the confirmed cases. From the time an individual comes into contact with the COVID-19 virus and contracts the disease, confirming the diagnosis can be a complex process. The length of time it can take for a case to be confirmed can be affected by incubation period, testing speed and report delay. The incubation period can vary, but most cases manifest themselves between 3 and 7 days after initial infection (Wang et al. 2020). The magnitude of the lag effect be even larger depending on the reporting variations in medical systems in different countries. A lag of 4 days was used to investigate the intervention policies across 33 provincial regions in China as a means of identifying both internal and external transmission effects (Oka et al. 2021). An optimal lag of 11 days was found to achieve the highest correlation between the mobility and COVID-19 growth rate ratios for a single all-county model in the United States (Badr et al. 2020) while Noland used 7-day and 14-day lags to estimate the reproduction number ${R}_{0}$ by a log-linear model (Noland 2021). For the modeling of multiple countries, it would appear that a generic 7-day lag can indicate that a 10 percentage point reduction in mobility is associated with a 0.04–0.07 reduction in ${R}_{0}$ (Bergman and Fishman 2020).

### The Google data

Using smart devices and digital transactions is the most rapid and convenient way to collect human mobility data for forecasting the COVID-19 pandemic (Chang et al. 2021; Guan et al. 2021; Leung et al. 2021). While the smart devices are GPS-tracked, the locations of the transaction data can be found at retail outlets, leisure facilities and other public amenities. Among the public datasets currently available, the Google COVID-19 Community Mobility Reports have been widely used for forecasting cases of infection and providing insights on how to use mobility characteristics efficiently (Wang and Yamamoto 2020; Bryant and Elofsson 2020; Achterberg et al. 2020; Schwabe et al. 2021; García-Cremades et al. 2021). Sufficient mobility records in both spatial and temporal dimensions enable the training of machine learning models that require large amount of data. Some research has adopted a number of statistical and machine learning models based on a recurrent neural network as well as an ensemble approach in order to predict trend changes in the 14-day cumulative incidence (García-Cremades et al. 2021). In this work, two datasets with similar training periods but different testing periods were used to compare the models. It is not surprising that the dataset with the shorter prediction period and stable trend outperformed the other one and demonstrated a greater predictability. The research also suggested that a 14-day cumulative incidence is predictive of mobility variables with the lag of seven days. In another work, a Network Inference-based Prediction Algorithm (NIPA), a combination of machine learning and phenomenological epidemiology, was proposed to predict the cumulative number of infected cases up to six days ahead (Achterberg et al. 2020). The research that has been done using network-based approaches has considered the interactions between different regions. Including a time-varying or static prior close to the true contact network may improve the performance of a NIPA across logistic function, sigmoid curves and LSTM. A mobility-marked Hawkes model was proposed for case prediction in the early stages of the pandemic (Schwabe et al. 2021). The study retooled to adopt a Hawkes process to capture the transmission dynamics. A mark, estimated by a regularized Poisson regression, was used to modulate the rate of infections and to account for the variations of ${R}_{0}$ across region and time. Finally, a correction procedure incorporated new cases seeded by people traveling between regions. With a forecasting period between 5 and 21 days, the model achieved outstanding performance when compared with six other baseline methods. Using the same data and the assumption of a 14-day incubation period, a recursive training-and-predicting process was conducted by characterizing the spatio-temporal dynamics (Wang and Yamamoto 2020). A Markov-Chain Monte-Carlo (MCMC) model was developed to estimate the spread of COVID-19 across a selection of 11 European countries and to further model the number of deaths from negative binomial distribution (Bryant and Elofsson 2020). For some unknown reason, when a 3-week forecast was reached, the model tended to overestimate the impact of non-pharmaceutical interventions in Sweden and Denmark. These modeling outcomes have provided us with ample resources for benchmarking new modeling methods and suggesting a range of policy implications.

### Indications

Various models and data sources have been identified in order to predict and manage the outbreak and spread of COVID-19 at different locations and at different geographical scales (Mohamadou et al. 2020; Zhang et al. 2021). It is still challenging to build a common modeling framework for COVID-19 case prediction that fits all regions and time periods. In addition to the inherent and complex dependencies in both spatial and temporal dimensions, individual behaviors may also affect the predictions. For example, people wearing a mask spread the virus to a smaller number of people and thus contribute to a flattening of the infection curve, even in a scenario without mobility restriction (Lima and Atman 2021). Travel restrictions may be less effective once the outbreak is more widespread, although they were highly effective measures in the early stages of the pandemic (Kraemer et al. 2020). However, the time boundary for distinguishing early and post-early stages is still unclear. Thus, any potential models need to be built separately in the study area and then compared across different scenarios before being deployed for policy making.

## Modeling method

### Models for count data

The number of daily confirmed COVID-19 cases can be modelled as count data from a Poisson (Zhang et al. 2020; Agosto and Giudici 2020) or a negative binomial distribution (Oztig and Askin 2020). The conditional Poisson model, denoted by ${Y}_{t}=y|{\mathcal{F}}_{t-1}\sim Poisson({\lambda }_{t})$, considers a set of historical factors, ${\mathcal{F}}_{t-1}$, at time $t-1$ to imply the distribution at time $t$:

1 $$\mathcal{P}\left({Y}_{t}=y|{\mathcal{F}}_{t-1}\right)=\frac{{\lambda }_{t}^{y}{e}^{-{\lambda }_{t}}}{y!}, \quad y=0, 1, 2,\dots $$

where ${Y}_{t}$ is a random variable with conditional expectation and variance $E\left({Y}_{t}=y|{\mathcal{F}}_{t-1}\right)=Var\left({Y}_{t}=y|{\mathcal{F}}_{t-1}\right)={\lambda }_{t}$. Supposing that the expectation can be characterized by ${Z}_{t}{\lambda }_{t}$ in a mixed Poisson model with a positive i.i.d. random variable ${Z}_{t}$ from the gamma distribution, the marginal distribution of ${\tilde{Y }}_{t}=y|{\mathcal{F}}_{t-1}$ then becomes a negative binomial distribution when $E({Z}_{t})=1$ and $Var\left({Z}_{t}\right)={\sigma }^{2}$ (Lawless 1987). A dispersion parameter $\phi =1/{\sigma }^{2}$ is introduced to formulate its probability mass function

2 $$\mathcal{P}\left({\tilde{Y }}_{t}=y|{\mathcal{F}}_{t-1}\right)=\frac{\Gamma (y+\phi )}{y!\Gamma (\phi )}{\left(\frac{{\lambda }_{t}}{{\phi +\lambda }_{t}}\right)}^{y}{\left(\frac{\phi }{{\phi +\lambda }_{t}}\right)}^{\phi }, y=0, 1, 2,\dots $$

denoted by ${\tilde{Y }}_{t}=y|{\mathcal{F}}_{t-1}\sim NB({\lambda }_{t}, \phi )$. In this setting, $E\left({\tilde{Y }}_{t}=y|{\mathcal{F}}_{t-1}\right)={\lambda }_{t}$ and $Var\left({\tilde{Y }}_{t}=y|{\mathcal{F}}_{t-1}\right)={\lambda }_{t}+{\lambda }_{t}^{2}/\phi$, which indicates that the Poisson variable is a limiting case when ${\sigma }^{2}\to 0$.

### Autoregressive model

In addition to the distributional features, the autoregressive relationship is also reflected in the time series data. In this paper, the Poisson autoregression is built upon an integer-valued generalized autoregressive conditional heteroskedastic (INGARCH) approach to linearly regress on a process of lagged values and conditional means (Ferland et al. 2006; Fokianos et al. 2009):

3 $${\lambda }_{t}={\beta }_{0}+\sum_{i=1}^{p}{\gamma }_{i}{Y}_{t-i}+\sum_{j=1}^{q}{\delta }_{j}{\lambda }_{t-j},$$

where ${\beta }_{0}$, ${\gamma }_{i}$ and ${\delta }_{j}$ are parameters to be estimated. The model facilitates the selection of lagged periods and the capture of seasonal effects by determining the values of ${\gamma }_{i}$ and ${\delta }_{j}$. One of the limitations for using INGARCH is that the inclusion of time varying covariates can only provide positive effects. Negative effects lead to unreasonable regressors for count variables. To allow for negative effects, this work thus adopts a more general modeling form of conditional count time series data under the framework of a generalized linear model (GLM) (Nelder and Wedderburn 1972; McCullagh and Nelder 1989), but extends this GLM by regressing on past terms such as ${Y}_{t-i}$ and ${\lambda }_{t-j}$ in Eq. (3) (Liboschik et al. 2017). The *autoregressive count data* (ACD) model used in this paper is:

4 $$g\left({\lambda }_{t}\right)={\beta }_{0}+\sum_{i=1}^{p}{\gamma }_{i}h\left({Y}_{t-i}\right)+\sum_{j=1}^{q}{\delta }_{j}g\left({\lambda }_{t-j}\right)+{{\varvec{\eta}}}^{\mathrm{\top }}{{\varvec{X}}}_{t},$$

where $g: {\mathbb{R}}^{+}\to {\mathbb{R}}$ is a link function that maps the expectation to a linear regressor and relaxes the positive constraint of the regressor in Eq. (3). The function $h: {\mathbb{N}}_{0}\to {\mathbb{R}}$ ensures that the lagged values are transformed to real values. ${\varvec{\eta}}=({\eta }_{1}, {\eta }_{2},\boldsymbol{ }\dots ,\boldsymbol{ }{\eta }_{m}{)}^{\mathrm{\top }}$ is another set of parameters on the covariate vector ${{\varvec{X}}}_{t}$. Similar to the GLM setting, the ACD model gives consistent maximum likelihood estimators when $g\left(x\right)=\mathrm{log}x$ and $h\left(x\right)=\mathrm{log}(x+1)$ (Fokianos and Tjøstheim 2011).

When an intervention, such as the introduction of a new travel policy related to COVID-19, occurs prior to $t$, the magnitude of its effect, $\xi {\Delta }^{t-\tau }1(t\ge \tau )$, can be added, where $\xi$ is the parameter, $\Delta \in \left[\mathrm{0,1}\right]$ determines the decay rate of the intervention effect and $\tau$ is the time of occurrence. Specifically, when $\Delta =0$, the intervention effect only exists at the time of its occurrence; when $0<\Delta <1$, the effect decays exponentially; and when $\Delta =1$, a constant effect persists after its occurrence until the end of the study period. $1$ is an indicator taking value 1 for $t\ge \tau$ and 0 for $t<\tau$. When multiple interventions generate effects on ${\lambda }_{t}$ for $0<\Delta \le 1$, a linear superposition of $r$, such interventions will form an additional component for the ACD model:

5 $$g\left({\lambda }_{t}\right)={\beta }_{0}+\sum_{i=1}^{p}{\gamma }_{i}h\left({Y}_{t-i}\right)+\sum_{j=1}^{q}{\delta }_{j}g\left({\lambda }_{t-j}\right)+{{\varvec{\eta}}}^{\mathrm{\top }}{{\varvec{X}}}_{t}+\sum_{k=1}^{r}{\zeta }_{k}{\Delta }^{t-{\tau }_{k}}1(t\ge {\tau }_{k}),$$

where ${\zeta }_{k}$ is a set of parameters reflecting the power of intervention at ${\tau }_{k}$. Thus, ${\mathcal{F}}_{t-1}$ is a collection of {${Y}_{t-1}$, ${Y}_{t-2}$, …, ${Y}_{1}$, ${\lambda }_{t-1}$, ${\lambda }_{t-2}$, …, ${\lambda }_{1}$, ${{\varvec{X}}}_{t}$, ${{\varvec{X}}}_{t-1}$, …, ${{\varvec{X}}}_{1}$, ${\Delta }^{t-{\tau }_{r}}$, …, ${\Delta }^{t-{\tau }_{1}}$}. The parameters to be estimated will be ${\varvec{\theta}}={({\beta }_{0}, {\gamma }_{1}, \dots , {\gamma }_{p}, {\delta }_{1}, \dots , {\delta }_{q}, {\eta }_{1},\boldsymbol{ }\dots ,\boldsymbol{ }{\eta }_{m}, {\zeta }_{1}, \dots , {\zeta }_{r})}^{\mathrm{\top }}$. Some of the elements in ${\varvec{\theta}}$ will equal 0 if the corresponding factors are not supposed to have an effect on ${\lambda }_{t}$. For the logarithm link $g$, constraints $\left|{\beta }_{0}\right|, \left|{\gamma }_{1}\right|, \dots , \left|{\gamma }_{p}\right|, \left|{\delta }_{1}\right|, \dots , \left|{\delta }_{q}\right|<1$ and $\left|\sum_{i=1}^{p}{\gamma }_{i}+\sum_{j=1}^{q}{\delta }_{j}\right|<1$ are further introduced (Liboschik et al. 2017). The dispersion parameter $\phi$ in Eq. (2) will be discussed and estimated separately in next section.

### Quasi-likelihood and parameter estimation

It is not uncommon for count data to be overdispersed, meaning that the variance can be larger than its mean value. Investigation into the variance-mean relationship can help with model selection (Ver Hoef and Boveng 2007). For a Poisson model, for example, it might be too strict to have an identical mean and variance. One practical way of modeling is to relax the assumption on the random component to allow for the function $\nu V\left({\lambda }_{t}\right)=Var\left({Y}_{t}|{\mathcal{F}}_{t-1}\right)$ to represent the randomness instead of characterizing an exponential distribution, where $\nu$ is the dispersion parameter and $V\left({\lambda }_{t}\right)={\lambda }_{t}$ implies the Poisson model. This relaxation leads to a more general expression of the likelihood function, that is, a *quasi-likelihood* (McCullagh 1983).

The construction of quasi-likelihood is based on a quasi-score function $S(\lambda )$ that is analogous to the score function of the ordinary likelihood. If we let

6 $$S\left(\lambda \right)=\frac{Y-\lambda }{\nu V\left(\lambda \right)},$$

it can be easily determined that $E\left(S\right)=0$ and $Var\left(S\right)=\frac{1}{\nu V(\lambda )}=-E\left(S{^{\prime}}\right)$. Thus, the quasi-likelihood function $Q(Y;\lambda )$ can be defined as (Wedderburn 1974):

7 $$ Q\left( {Y;\lambda } \right) = \int_{{ - \infty }}^{\lambda } {\frac{{Y - u}}{{\nu V\left( u \right)}}} du + f\left( Y \right), $$

where $f\left(Y\right)$ is a real-value function of $Y$. The dispersion parameter $\nu$ ($\phi$ for the negative binomial in this paper) usually does not affect the estimation of ${\varvec{\theta}}$ and can be estimated in a separate step. For the negative binomial distribution, one of the approaches is to approach is to consider the moment estimation and solve Eq. (8) (Lawless 1987; Christou and Fokianos 2014):

8 $$\sum_{t=1}^{n}\frac{{\left({\tilde{Y }}_{t}-{\widehat{\lambda }}_{t}\right)}^{2}}{{\widehat{\lambda }}_{t}(1+{\widehat{\lambda }}_{t}/\phi )}=n-(p+q+m+r+1),$$

where given the estimation of ${\varvec{\theta}}$, ${\widehat{\lambda }}_{t}={\lambda }_{t}(\widehat{{\varvec{\theta}}})$ is the fitted value of ${\lambda }_{t}$ and $n$ is the sample size.

In GLM, a quasi-Poisson model represents a class of distributions rather than a single quasi-Poisson distribution. The negative binomial model, one of the most convenient examples of the mixed Poisson process introduced in Sect. 3.1, is usually employed to account for the extra variations in the Poisson model (Lawless 1987). Despite the fact that overdispersion is a more frequently observed feature (Zhu 2012), quasi-Poisson can also be used for modeling underdispersion. Thus, the conditional Poisson-like quasi-log-likelihood, also shared by the negative binomial model, for the ACD model is specified by plugging $V\left(u\right)=u$ and $f\left(Y\right)=0$ into Eq. (7):

9 $$l\left({\varvec{\theta}}\right)=Q\left({y}_{t};{\lambda }_{t}({\varvec{\theta}})\right)=\sum_{t=1}^{n}\left({y}_{t}\mathrm{ln}\left({\lambda }_{t}\left({\varvec{\theta}}\right)\right)-{\lambda }_{t}({\varvec{\theta}})\right).$$

It has been shown in Liboschik et al. (2017) that $\partial {\lambda }_{t}\left({\varvec{\theta}}\right)/\partial{\varvec{\theta}}$ can be obtained recursively and thus come to form the differential calculations in the score function $\partial l\left({\varvec{\theta}}\right)/\partial{\varvec{\theta}}$. The quasi-likelihood estimator is thus expressed as:

10 $$\widehat{{\varvec{\theta}}}={\mathrm{arg}max}_{{\varvec{\theta}}}l\left({\varvec{\theta}}\right).$$

To solve Eq. (10), the first step is to use an adaptive barrier algorithm (Lange 2010) to enforce the constraints of the non-intervention parameters. A quasi-Newton BFGS is then applied for optimization without constraints (Broyden 1970; Goldfarb 1970; Shanno 1970; Fletcher 1970).

## Data and workflow

In this paper we illustrate the relationship between mobility and COVID-19 transmission by examining the case of Sweden, a country with the fifth largest land area in Europe and one characterized as having a low population density. Low population density implies there is a relatively large distance between people compared to other European countries and that the extent of unobserved close contact is also low.

### Mobility data

Substantial efforts have been made to create data sets that reflect mobility change since the outbreak of the COVID-19 pandemic, where starting points between January 2020 and February 2020 have been selected to provide a baseline (Hale et al. 2020; Aktay et al. 2020; Apple. 2021). The baselines enable the user to compare the effect of policy interventions or mobility changes during the COVID-19 period. The mobility data usually reflects where people move from and to and how long they stay at their destinations. Due to fact that many travelers in our study region significantly changed their mobility patterns by abandoning public transport after the pandemic broke out (Jenelius and Cebecauer 2020), our assumption is that the highest risk of COVID-19 transmission occurs, not when people travel by public transport, but when they remain in a location for a period of time. In this paper, the mobility data collected by Google (Google 2021) will be divided into six different types of locations that represent similar categories of mobility behavior. These categories are *retail and recreation* (e.g., restaurants, cafes, museums, libraries, and movie theaters), *groceries and pharmacies* (e.g., food warehouses, farmers markets, specialty food shops, and pharmacies), *parks* (e.g., local parks, national parks, public beaches), *transit stations* (e.g., subway, bus, and train stations), *workplaces*, and *residential locations*.

The data presents the daily change of visits and length of stay, tracked by mobile devices, for each location category in Sweden from February 15, 2020 and compares it to the baseline days. The relative change in percentage is reported for Sweden as a whole as well as for each region and municipality. For the smaller municipalities without sufficient observations to aggregate, however, missing values are reported thus making it pointless to interpolate synthetic values. On the regional and national levels, the proportion of missing values is marginal for most of the categories. The baseline days are taken from five weeks at the beginning of 2020, where seven median values for each day of a typical week are selected as the baseline values.

### Confirmed cases of COVID-19 in Sweden

**Fig. 1:**
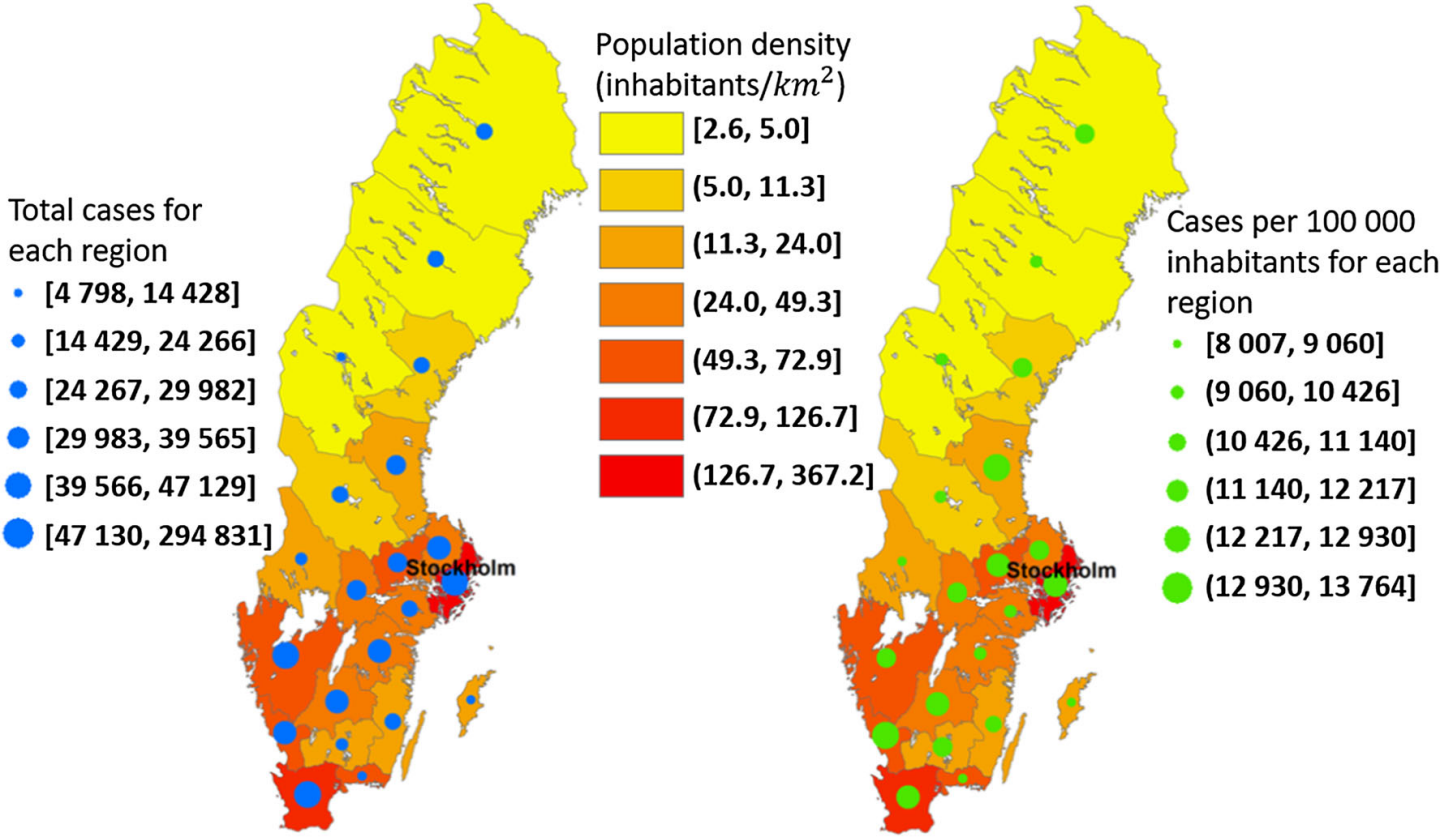
Regions of Sweden and confirmed cases

There are 21 regions (administrative counties) in Sweden. The majority of daily moving activity in Sweden occurs within these regional borders because the places that most people visit each day, such as schools and hospitals, are regionally organized and tend to be located in the vicinity of residential areas and workplaces. The data produced by the Swedish Public Health Agency provides a daily report of the total number of confirmed cases of COVID-19 for each region (The Public Health Agency of Sweden 2021). To control for the effect of regional population differences, we also consider the relative number of confirmed cases per hundred thousand inhabitants. A map of the 21 Swedish regions showing both calculations is shown in Fig. 1.

In Fig. 1, regional population density is also shown because it is an important local feature that may have a latent impact on modeling performance. Figure 1 shows, per region, the total number of confirmed COVID-19 cases and cases per thousand inhabitants from the date the first Swedish case was officially reported, February 4, 2020, to the date of writing, December 7, 2021. It is no surprise that the regions with the highest population density have the most confirmed cases. Stockholm, Sweden’s capital city and the region with the country’s highest population density, for example, has also recorded the highest number of cases, over 295,000. For the relative figures in the right panel of Fig. 1, population density does not seem to be an obvious determinant of COVID-19 frequency.

### Workflow

**Fig. 2:**
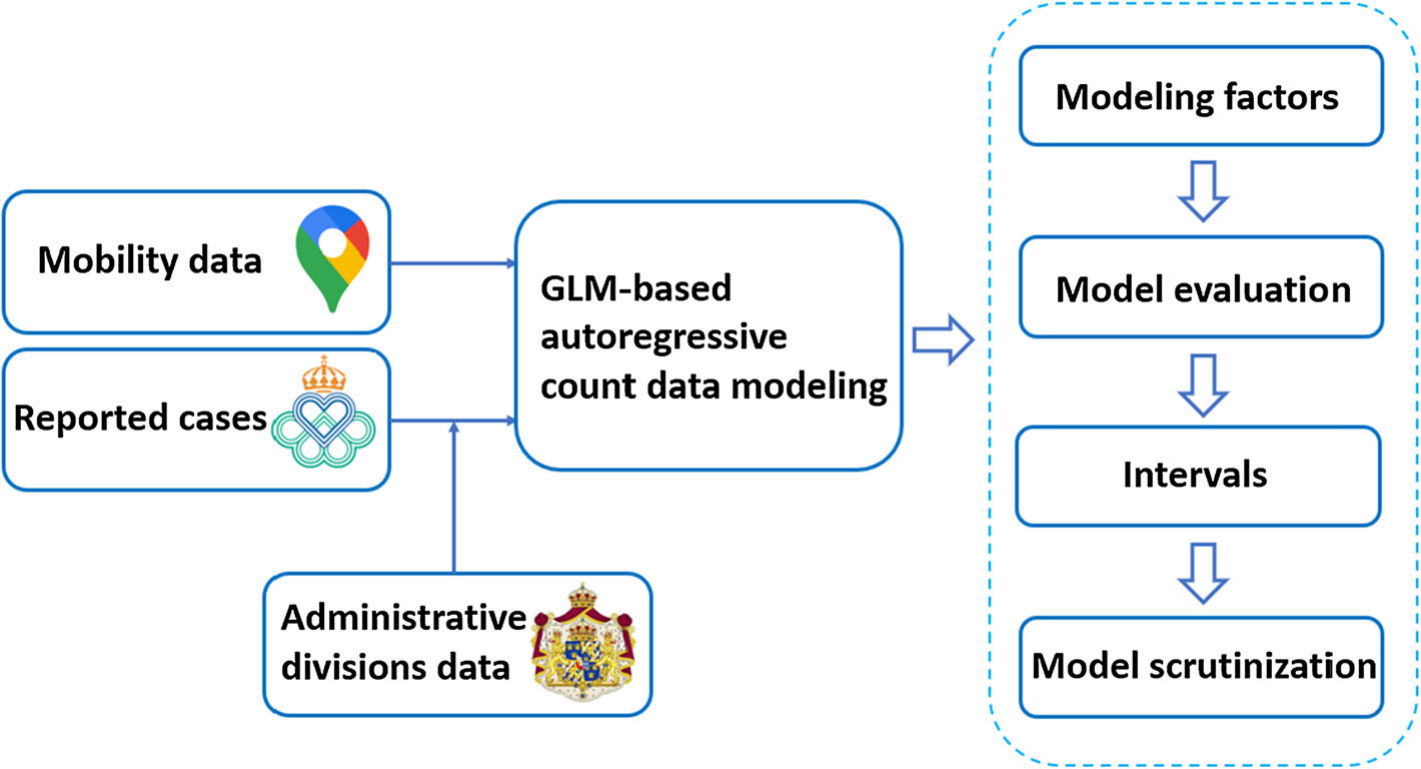
Modeling workflow

Based on the modeling framework and data described in previous sections, the workflow of the paper is summarized in Fig. 2. The categories in the mobility data are covariates that were fixed in both the modeling and prediction stages. The COVID-19 data was fused with the administrative division data and was treated as a random variable. In order to evaluate the model’s performance, five factors that might generate discrepancies in the modeling were considered. A holdout set was also selected to evaluate the model’s performance. Point prediction was computed for all possible scenarios and intervals from the best models were taken to further examine the coverage. Finally, model scrutinization was carried out to investigate the statistical meaning of the models where intervention was included.

## Results

In this section we present the five factors (and their values) that allowed us to observe and summarize a wide range of prediction results from multidimensional angles. Both point predictions and interval coverages were used to assess the performance of the models with different scenarios. After including interventions, the analytical results provided a number of insights into the statistical features.

### Factors

The study period selected for modeling was January 1, 2021 to December 7, 2021, where the last two weeks in the series were taken to form a holdout set for testing the predictions from different models. The five factors that were used to evaluate the model’s performance in Fig. 2 were lag, region, model, ${Y}_{t-i}$ and ${\lambda }_{t-i}$. The number of elements and the specific values for each factor are presented in Table 1.

**Table 1:** Factors considered in modeling

Factors	No. of elements	Values
Lag (days)	11	2–12
Region^a^	21	20 regions plus whole country
${Y}_{t-i}$	2	$i=1$ and $i=7$
${\lambda }_{t-i}$	2	$i=1$ and $i=7$
Model	24	I, II , …, XII for both Poisson and neg. bin

^a^1-whole country, 2-Blekinge, 3-Dalarna, 4-Gävleborg, 5-Halland, 6-Jämtland, 7-Jönköping, 8-Kalmar, 9-Kronoberg, 10-Norrbotten, 11-Örebro, 12-Östergötland, 13-Skåne, 14-Södermanland, 15-Stockholm, 16-Uppsala, 17-Värmland, 18-Västerbotten, 19-Västernorrland, 20-Västmanland, 21-Västra Götaland

Lag refers to the number of days prior to the COVID-19 report date that will be selected for modeling the mobility data. COVID-19 has a 4 to 9-day incubation period before symptoms are perceived (Tindale et al. 2020). When mobility is added to the mix, the lag for accurate prediction can rise to 12 days. (Badr et al. 2020). It can also take several days for test results to be reported. Due to these delays, and so as not to miss any potential features, we studied a range of 2–12 days. Region refers to the geographical division upon which the model is built, in this case the Swedish administrative county. Of the 21 regions included in our models, the region Gotland did not have complete mobility data for the whole of the study period. Thus, its data was disregarded and the models were built around the 20 remaining regions. We also included whole Sweden as an additional region for comparison.

The two types of autoregressive terms, ${Y}_{t-i}$ and ${\lambda }_{t-i}$, were used to determine how the number of confirmed cased for any given day were related to the previous observations and their expected values or seasonal effects. Since the number of possible combinations was too great to calculate, we only considered three of them: the most recent ones: 1) $\langle {Y}_{t-1}, {\lambda }_{t-1}\rangle$, and one with weekly seasonality: 2) $\langle {Y}_{t-1}, {\lambda }_{t-7}\rangle$ and 3) $\langle {Y}_{t-7}, {\lambda }_{t-1}\rangle$. This was because the baseline days were chosen from a period prior to the onset of COVID-19 and represent a typical week. The variation in models concerns the specification of different covariates, how ${Y}_{t}$ is measured and what distribution is assumed. For the covariates, missing values for the variable *parks* were found for some of the regions and thus this variable could not be included in our tests. The remaining five variables were included as covariates, both individually and all together, thus making six different models. For ${Y}_{t}$, two measurements—daily total confirmed cases and daily confirmed cases per hundred thousand inhabitants—were used for modeling. The latter measurement was given a relative value to eliminate the influence of population size. Thus, the Poisson model with six types of covariates to model total cases were denoted as I, II, …, VI, respectively. Those modeling relative values were denoted as VII, VIII, …, XII, respectively. The settings were replicated for negative binomial distribution. Since both distributions share the same likelihood function, as shown in Eq. (9), the point estimators of the parameters were the same. The difference between them can be seen in the estimation of standard errors and the intervals of predicted values. In conclusion, while the factors considered here may not be exhaustive, they are sufficient to provide an overview of a general evaluation framework.

All of the factors taken together formed $11\times 21\times 3\times 24=16 632$ models due to the large number of combinations of regions and models. Although models including entire study regions and all covariates were studied, it is still appealing to compare models with every single region and covariate. There are two main reasons for doing so. First, there are significantly different features between the regions, e.g., population density, mobility level, and social structure. The selected model, which is tailored for a single region and may not be appropriate to another region, will facilitate local management of medical resource allocation and policy making. Second, we use the open data collected by Google to illustrate the model selection in this paper. However, collecting all covariates may be infeasible or expensive for regional studies. If a single covariate can lead to accurate prediction models rather than using all covariates, the modeling method can be efficiently conducted thanks to the lowered burden in data collection.

### Model evaluation

**Fig. 3:**
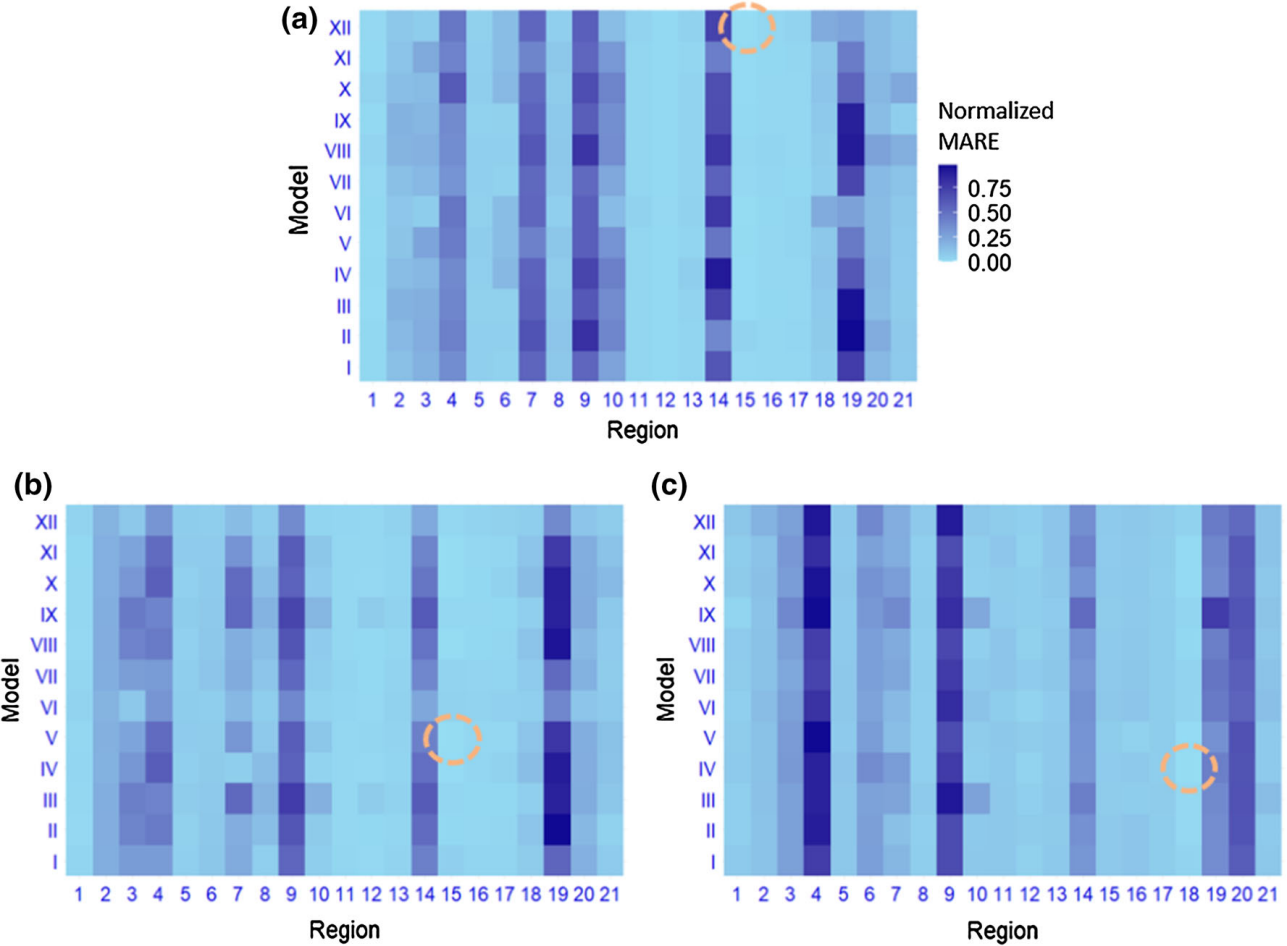
Normalized MARE for three example scenarios where the best models were found: **a** $\langle {Y}_{t-1}, {\lambda }_{t-1}\rangle$ and lag 8; **b** $\langle {Y}_{t-1}, {\lambda }_{t-7}\rangle$ and lag 5; **c** $\langle {Y}_{t-7}, {\lambda }_{t-1}\rangle$ and lag 10

All models were evaluated on the holdout set, which implies that each model could generate additional 14-day predicted values. The accuracy was defined by the mean absolute relative errors (MARE):

11 $$MARE=\sum_{t=1}^{14}\frac{\left|\widehat{{Y}_{t}}-{Y}_{t}\right|}{{Y}_{t}}$$

for each model. Since the case number 0 is not defined for MARE, it was excluded in the evaluation. We will show that this caused only negligible effects on the global evaluation. For each lag number and combination of autoregressive terms, the best MARE was selected to indicate which model-region among all of them was the most reliable. In Fig. 3a, for example, the MAREs of the scenario $\langle {Y}_{t-1}, {\lambda }_{t-1}\rangle$ and a lag of 8 for all regions and models were normalized and compared. The outcome produced by region 15 and model XII gave the lowest MARE. Similarly, the configuration for the scenario $\langle {Y}_{t-1}, {\lambda }_{t-7}\rangle$ and lag 5 was presented in Fig. 3b and $\langle {Y}_{t-7}, {\lambda }_{t-1}\rangle$ and lag 10 in Fig. 3c. From Fig. 3, it seems that model performance tends to have less variance within regions than between regions.

In order to investigate how the top-ranked model-region combinations could be consistently obtained for each scenario, an overall evaluation was conducted and is presented in Table 2. It should be noted that Table 2 gives the results of the top two model-region combinations as well as the MAREs that applies to both distributions. In general, across different scenarios, ten out of twelve models achieved the lowest MAREs. For none of the scenarios did Model Ior VII perform best, thus indicating that only measuring mobility change in retail and recreation locations by themselves does not provide reliable predictions. Model III and IV showed good accuracies for most lags given $\langle {Y}_{t-1}, {\lambda }_{t-1}\rangle$. Nevertheless, XII (with a lag 8 and 2) ranked in the top two for this setting. Similarly, model V (lag 5) and II (lag 6) achieved the lowest errors given $\langle {Y}_{t-1}, {\lambda }_{t-7}\rangle$ while model IV (lag 10 and 2) outperformed the other models for $\langle {Y}_{t-7}, {\lambda }_{t-1}\rangle$. With regard to the regions, almost all of the scenarios for $\langle {Y}_{t-1}, {\lambda }_{t-1}\rangle$ and $\langle {Y}_{t-1}, {\lambda }_{t-7}\rangle$ ended up with selecting the most populous region, 15-Stockholm. Only two scenarios that ranked second (lag 7 and 9 for $\langle {Y}_{t-1}, {\lambda }_{t-7}\rangle$) ended up with selecting region 1-whole country. For $\langle {Y}_{t-7}, {\lambda }_{t-1}\rangle$, the best models for all 22 scenarios were emerged from the data from region 18-Västerbotten, a region characterized by its low population density. The following analysis, therefore, will focus on six of the models, the top 2 for each autoregressive setting.

**Table 2:** MARE for each lag number and combination of autoregressive terms

Lag No	$\langle {Y}_{t-1}, {\lambda }_{t-1}\rangle$		$\langle {Y}_{t-1}, {\lambda }_{t-7}\rangle$		$\langle {Y}_{t-7}, {\lambda }_{t-1}\rangle$	
	1st	2nd	1st	2nd	1st	2nd
2	**XII (0.189)**	V (0.196)	IX (0.197)	III (0.202)	IV** (0.133)**	V (0.147)
3	III (0.195)	V (0.196)	XI (0.202)	X (0.210)	X (0.138)	IV (0.138)
4	III (0.196)	IV (0.200)	X (0.197)	V (0.206)	X (0.160)	IV (0.168)
5	III (0.202)	IV (0.202)	V** (0.177)**	XI (0.187)	V (0.158)	XI (0.159)
6	III (0.204)	IV (0.210)	II** (0.186)**	VIII (0.191)	X (0.149)	XI (0.152)
7	III (0.209)	IV (0.210)	VIII (0.205)	IV (0.207)*	VIII (0.157)	II (0.160)
8	**XII (0.175)**	VI (0.191)	XI (0.188)	XII (0.200)	IV (0.136)	V (0.138)
9	XII (0.203)	III (0.211)	IX (0.203)	XII (0.232)*	V (0.148)	II (0.162)
10	III (0.208)	IV (0.209)	XI (0.199)	X (0.206)	IV** (0.130)**	V (0.139)
11	IV (0.203)	III (0.207)	X (0.196)	IV (0.203)	X (0.150)	IV (0.158)
12	IV (0.201)	III (0.212)	X (0.188)	V (0.190)	IV (0.136)	X (0.141)

*The whole country as the best region

### Intervals

In the 1-step-ahead prediction of ${\widehat{Y}}_{t+1}$, the expected value ${\lambda }_{t+1}$ was estimated, first of all, by the exponential operation and plugging in of the covariates at time $t+1$ using $\widehat{{\varvec{\theta}}}$:

12 $${\widehat{\lambda }}_{t+1}=exp\left\{{\lambda }_{t+1}\left(\widehat{{\varvec{\theta}}}\right)\right\}.$$

**Fig. 4:**
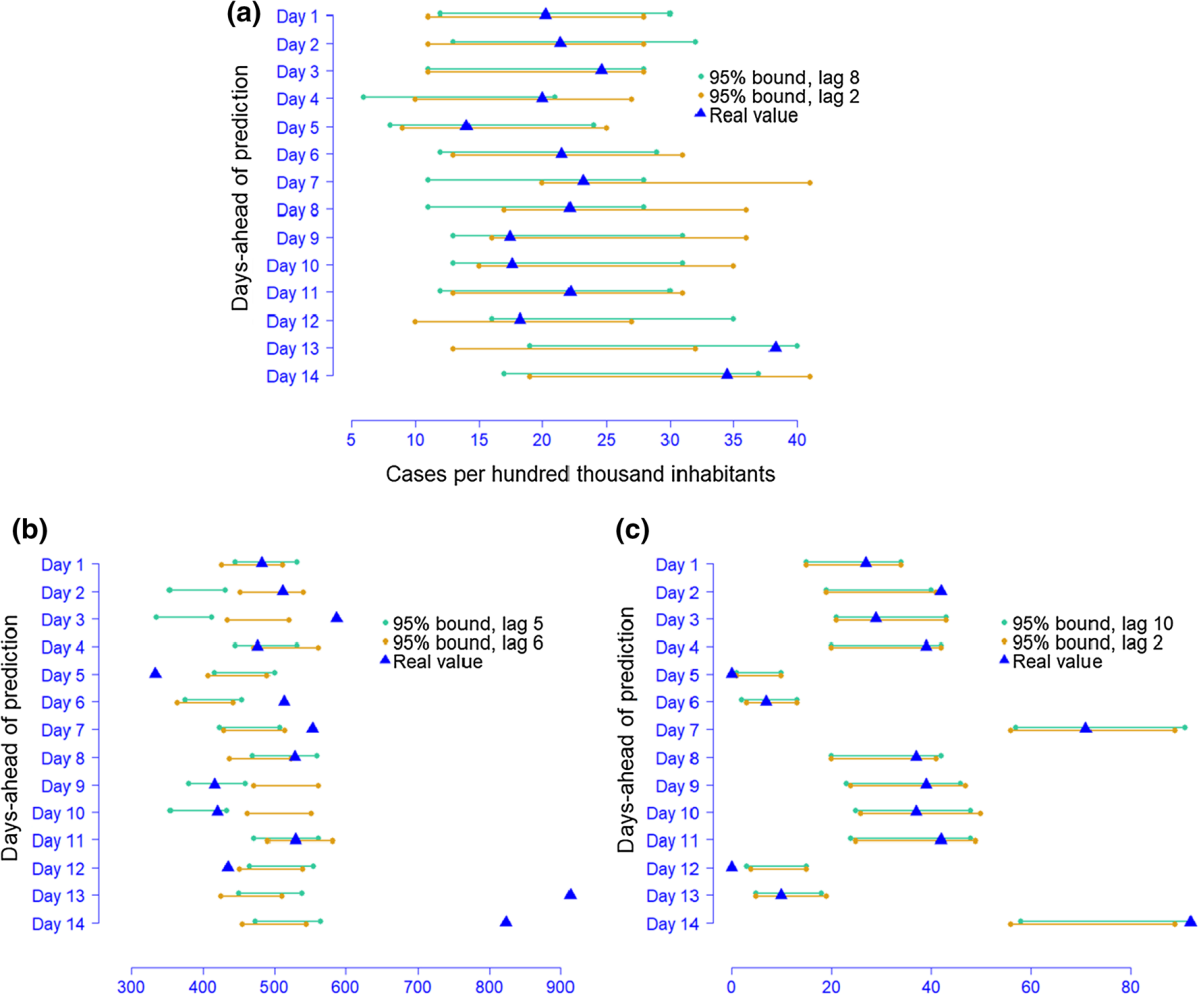
95% intervals for top two models for selected scenarios: **a** $\langle {Y}_{t-1}, {\lambda }_{t-1}\rangle$; **b** $\langle {Y}_{t-1}, {\lambda }_{t-7}\rangle$; **c** $\langle {Y}_{t-7}, {\lambda }_{t-1}\rangle$

To have a minimal mean square error, ${\widehat{Y}}_{t+1}$ took the value ${\widehat{\lambda }}_{t+1}$. Both of them, as well as the covariates at time $t+2$, formed the conditional factor ${\mathcal{F}}_{t+1}$ to get the 2-step-ahead prediction of ${\widehat{\lambda }}_{t+2}$ and ${\widehat{Y}}_{t+2}$. At this point, we did not use any real observations to serve as past information for the whole predictions so that the model uncertainty was close to reality. The update continued until fourteen days were obtained. Meanwhile, we estimated 95% confidence intervals since they reflected the ability of a model to cover real values. The variances were given in Sect. 3.1. Illustrative intervals for the six Poisson models are shown in Fig. 4. Intervals for negative binomial models were also computed, but they were less informative due to larger variance.

In Fig. 4a, the intervals of the lag 8 model covered all real fourteen day-ahead values in the holdout set and the lag 2 model missed covering only one. This result accords with those in Table 2. In Fig. 4b, since the intervals of the globally best model could still fail to include some values, the information provided was thus deemed to be unreliable. Figure 4c resembles Fig. 4a because both lag 10 and lag 2 models were able to capture the variations. Model IV, however, was the most suitable for modeling the total number of confirmed cases. Notably, 0 cases were reported on day 5 and day 12. Models for these two days were able to cover or be very close to covering 0, implying that the model performance is resistant to extreme values. Overall, model XII at lag 8 and IV at lag 10 are trustable for modeling densely and sparsely populated regions, respectively. It is worth noting that the accuracy does not decline as the number of predicted days increases. However, there is no model that can better handle the autoregressive terms $\langle {Y}_{t-1}, {\lambda }_{t-7}\rangle$ than another.

### Model scrutinization

**Fig. 5:**
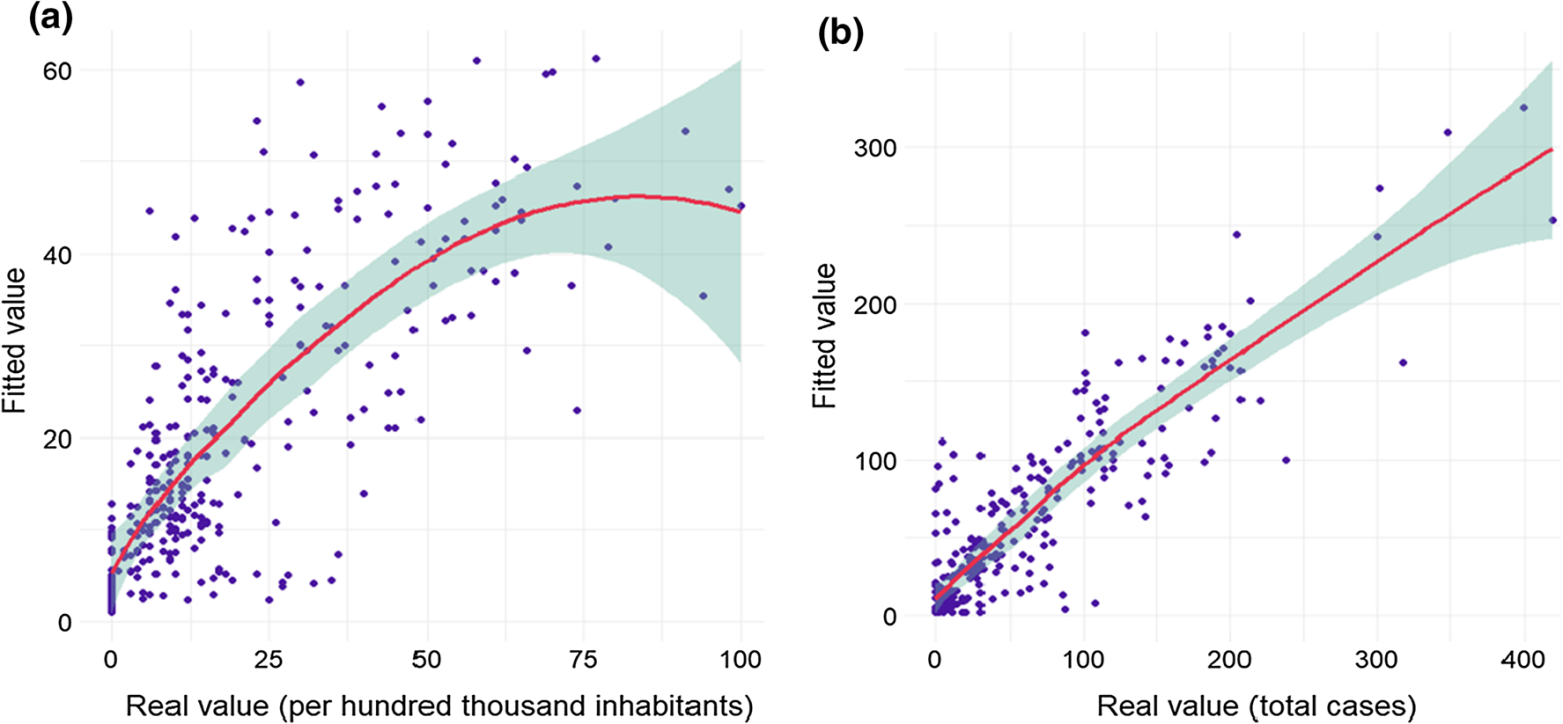
Fitted values against real values from models with interventions **a** model XII at lag 8 given $\langle {Y}_{t-1}, {\lambda }_{t-1}\rangle$; **b** model IV at lag 10 given $\langle {Y}_{t-7}, {\lambda }_{t-1}\rangle$

These two identified models—model XII at lag 8 given $\langle {Y}_{t-1}, {\lambda }_{t-1}\rangle$ and model IV at lag 10 given $\langle {Y}_{t-7}, {\lambda }_{t-1}\rangle$—were further investigated by adding an intervention and then comparing the two distributions. The intervention was introduced on September 29, 2021 because in Sweden on that date many of the pandemic-related restrictions, such as the low maximum attendance numbers for private and public gatherings and events, were removed. Increased social activities could possibly change the mobility pattern. The decay rate $\Delta$ was set as 0.5. The MAREs, however, were found to be slightly smaller than those without interventions (Table 3). The models with interventions, therefore, are recommended for making inferences. Fitted values against respective real values were plotted in Fig. 5, where smoothed Loess intervals were also given. In both Fig. 5a and Fig. 5b, the Loess intervals start to become larger at about the 97% quantile. The fitted values tend to underestimate the large real values. In addition, the fitted values for model at lag 8 with intervention present a quadratic trend while a linear trend is found for model IV at lag 10 with intervention. From the perspective of modeling, both types of trends can be characterized in GLM.

**Table 3:** Model comparison with an intervention

Model	XII, lag 8	IV, lag 10
Autoregressive factors	$\langle {Y}_{t-1}, {\lambda }_{t-1}\rangle$	$\langle {Y}_{t-7}, {\lambda }_{t-1}\rangle$
Sig. parameters ($\alpha =0.05$, Poisson)	${\gamma }_{1}=0.595$; ${\delta }_{1}=-0.209$; ${\eta }_{ret.}=5.226$; ${\eta }_{pha.}=-2.892$; ${\eta }_{tra.}=-4.702$; ${\eta }_{w.p.}=3.294$;${\eta }_{res.}=11.956$	${\gamma }_{7}=0.845$; ${\eta }_{w.p.}=0.228$
Sig. parameters ($\alpha =0.05$, neg. bin.)	${\gamma }_{1}=0.595$; ${\eta }_{w.p.}=3.294$;${\eta }_{res.}=11.956$	${\gamma }_{7}=0.845$
$1/\phi$	1.333	4.282
Intervention date	September 29, 2021 (56 days to Day 1)	
$\Delta$	0.5	
MARE	0.174	0.128

Significant parameters, as well as the estimation of $1/\phi$ for both models, are also given at the 0.05 level in Table 3. While all parameters under a Poisson assumption are significant for model XII at lag 8, due to the larger variance, only ${\gamma }_{1}$, ${\eta }_{w.p.}$ and ${\eta }_{res.}$ are significant for negative binomial distribution. Therefore, we consider that the difference between the two distributions might filter out the variables that depend on others. For example, reduced mobility at bus and train stations could increase the number of people gathered at residential locations. Thus, a direct recommendation to practitioners when making predictions for dense population areas is to collect mobility data from various types of locations. If difficulties restrict the full collection of data, the mobility changes collected from residential locations should be primarily used for modeling. One of the reasons for this is that residential location, in comparison to other location categories, is particularly sensitive to the key transmission mechanism of the COVID-19, that is, the personal contacts between family members and neighbors.

For model IV at lag 10, recommended for modeling regions with low density populations, the values reported for one week before and mobility change at the workplace were the most significant. When the negative binomial distribution is used, the weekly correlation, which also regulates working patterns, should be the one adopted for inference.

Finally, we computed the difference between the average predictive cumulative distribution function (CDF), $\overline{G }\left(y\right)\doteq \underset{T\to \infty }{\mathrm{lim}}\left\{\frac{1}{T}\sum_{t=1}^{T}{G}_{t}(y)\right\}$, and the true data-generating CDF, $\overline{F }\left(y\right)\doteq \underset{T\to \infty }{\mathrm{lim}}\left\{\frac{1}{T}\sum_{t=1}^{T}{F}_{t}(y)\right\}$, of the threshold values $y$ to make a distributional comparison by the marginal calibration (Christou and Fokianos 2015), where ${G}_{t}(y)$ and ${F}_{t}(y)$ are CDFs of sequential time series variables. ${G}_{t}(y)$ is said to be marginally calibrated relative to ${F}_{t}(y)$ if $\overline{G }\left(y\right)=\overline{F }\left(y\right)$ for all $y\in {\mathbb{R}}$ (Gneiting et al. 2007). Thus, the difference between $\overline{G }\left(y\right)$ and $\overline{F }\left(y\right)$ reflects how close the predicted CDF from the empirical model is to the theoretical one.

**Fig. 6:**
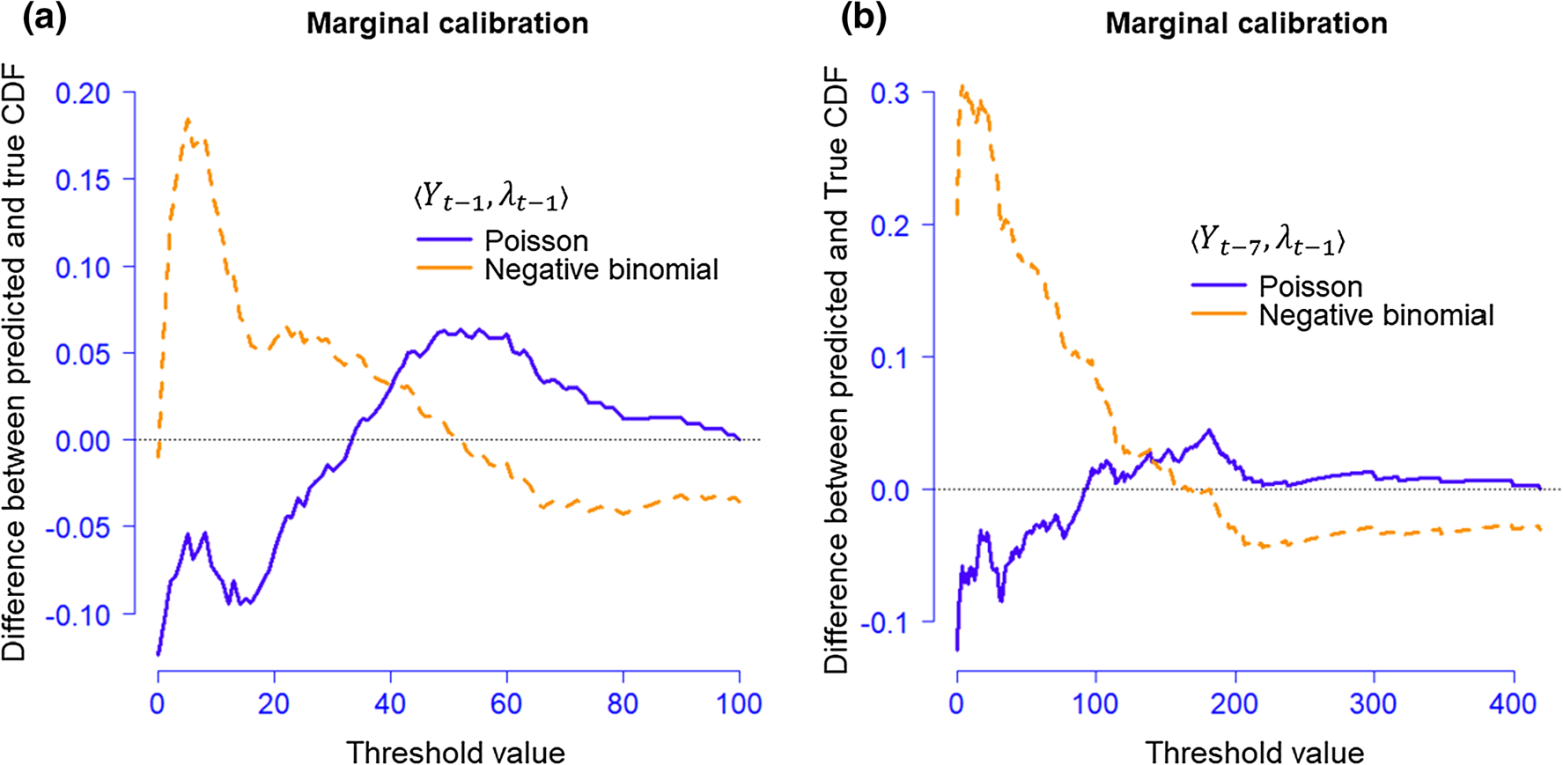
Marginal calibration **a** model XII at lag 8 given $\langle {Y}_{t-1}, {\lambda }_{t-1}\rangle$; **b** model IV at lag 10 given $\langle {Y}_{t-7}, {\lambda }_{t-1}\rangle$

Figure 6 provides a comparison of the two selected models. For both distributions in both models, the differences in CDFs are within $\pm 0.3$. For most threshold values, the Poisson models have the difference in CFD less than 0.1 for model XII and less than 0.05 for model IV. However, the difference given by the negative binomial distribution is relatively larger. For model IV, using Poisson assumption to model a set of values over 100 will largely satisfy the statistical assumptions. For the remaining sets of values, for asserting distributional features, the choice between the two distributions is inconclusive.

## Discussions

One of the fundamental assumptions of our method is that infected cases of COVID-19 in a region are not affected by another region. Although intra-region movement is most common way of individual mobility, inter-region movement cannot be neglected, especially for people living close to the border of a region. The lack of detailed individual moving data at a national scale hinders the statistical inference from including the cross-border effect. We believe that the inclusion of measuring the magnitude of cross-border movement will characterize the spatial interactions between regions more realistically. The measurements reflecting the proximity between regions can be adopted to estimate a correlation/covariance matrix. The matrix can be then decomposed to form random effects for each region by extending our modeling framework to a generalized linear mixed model with autoregressive count variables. Although data availability is a big obstacle for in-depth modeling, our proposed method can be quickly implemented by utilizing the assumption. Based on it, a pilot study with cross-border movement for a specific region can be conducted as a supplementary analysis, where, rather than national level, only regional or municipality level decision of data collection is required.

As for the modeling results, e.g., MARE and Fig. 5, there is still room to improve the accuracy of point predictions and fitted values, although the prediction at the 14-day-ahead scale provides favorable results compared the up-to-date studies. A direct strategy is to include more data at granular level to capture additional features in the modelling. However, acquiring such data, e.g., number of contacts to infected persons or individual risk level of infection, is not an easy task due to data privacy and its inherent immeasurability. As such, this paper presents a method by using a minimal cost in data collection to efficiently identify a way of model selection where interval coverages are acceptable.

## Conclusions

Since its first discovery in China back in December 2019, the COVID-19 pandemic has been responsible for at least 5.6 million deaths and has caused unfathomable adjustments to the world’s social and economic systems. Every country in the world has introduced some sort of policy to cut off the channels of transmission by controlling human mobility, through such measures as national lockdowns, tight restrictions on public assemblies, self-monitored quarantines and reductions and restrictions on international travel. Despite these restrictions, some degree of mobility is inevitable even just to ensure the meeting of daily needs. Effective epidemic prevention and control, therefore, must take mobility into account. This paper used an autoregressive count data model under the framework of a generalized linear model and with five categories of mobility data collected by Google to explore the construction of a model that could be adopted to predict reliable confirmed cases in 14 days.

Empirical modeling and evaluation of long-term data from Sweden shows that: (1) Mobility change in retail and recreational locations, groceries and pharmacies, transit stations, workplaces and residential locations is the better way to predict confirmed cases in relative numbers for a region with a high population density (367 inhabitants/km^2^ or higher, in this paper), where a lag of 8 days between the date of the observed mobility change and the reported cases is recommended in terms of interval coverage. Mobility in residential locations should be firstly collected with low availability of data. Past observations and conditional means one-period back may be included to provide autoregressive effects. (2) It can be concluded from Table 3 that, mobility change at workplaces as well as a lag of weekly confirmed cases, are considered to be the most relevant variables when predicting total confirmed cases in sparsely populated regions (5 inhabitants/km^2^ or lower, in this paper) for Poisson model. In the context of negative binomial model, the mobility change at workplaces does not seem to be influential. In this case, a lag of 10 days is recommended, which is consistent with the results studied in other countries. The model used in these cases is also robust when the value zero is reported. (3) Policy interventions can be included when modeling long-term pandemics. In this paper, the effect of the lifting of the ban on public gatherings was used as a national intervention in the modeling with the result that the prediction accuracies were slightly improved. Although both Poisson and negative binomial distributions provided identical point predictions to confirmed cases, the interval estimation of the Poisson model was more compact. Underestimates in large magnitude were only detected for a small proportion (3%) from the fitted values. With regard to parameter estimation, negative binomial models tend to lead to fewer significant parameters due to the introduction of additional variance. This model may be used when the collection of mobility data is too expensive. (4) A marginal calibration shows, from the statistical perspective, that the choice of distribution for count data is inconclusive except for the values over 100, where both Poisson and negative binomial models can be assumed when making the inference.

Addressing other limitations of this paper in future research may yield additional insights in epidemic modeling. First, as the development of communications technology and the internet of things continues apace, the inclusion of GPS-tracked mobility data during travel will add a new dimension to modeling, but will require more free accesses to the relevant databases. Second, it is still unknown how the models work in different environmental settings. Modeling outcomes from other countries could provide complementary information that would strengthen decision support. Third, other types of data-driven methods that can simultaneously handle time dependency, spatial correlation and count data might be alternative ways of modeling disease transmission.
